# Distribution Heterogeneity of Muscle Spindles Across Skeletal Muscles of Lower Extremities in C57BL/6 Mice

**DOI:** 10.3389/fnana.2022.838951

**Published:** 2022-03-17

**Authors:** Wenxi Lian, Fei Hao, Peng Hao, Wen Zhao, Yudan Gao, Jia-Sheng Rao, Hongmei Duan, Zhaoyang Yang, Xiaoguang Li

**Affiliations:** ^1^Beijing Key Laboratory for Biomaterials and Neural Regeneration, Beijing Advanced Innovation Center for Biomedical Engineering, School of Biological Science and Medical Engineering, Beihang University, Beijing, China; ^2^Department of Neurobiology, Capital Medical University, Beijing, China

**Keywords:** muscle spindle, distribution, heterogeneity, intrafusal fibers, dissymmetry

## Abstract

Muscle spindles, an important proprioceptor scattered in the skeletal muscle, participate in maintaining muscle tension and the fine regulation of random movement. Although muscle spindles exist in all skeletal muscles, explanations about the distribution and morphology of muscle spindles remain lacking for the indetermination of spindle location across muscles. In this study, traditional time-consuming histochemical technology was utilized to determine the muscle spindle anatomical and morphological characteristics in the lower extremity skeletal muscle in C57BL/6 mice. The relative distance from spindles to nerve-entry points varied from muscles in the ventral-dorsal direction, in which spindles in the lateral of gastrocnemius were not considered to be close to its nerve-entry point. In the longitudinal pattern, the domain with the highest abundance of spindles corresponded to the nerve-entry point, excluding the tibialis anterior. Spindles are mainly concentrated at the middle and rostral domain in all muscles. The results suggest a heterogeneity of the distribution of spindles in different muscles, but the distribution trend generally follows the location pattern of the nerve-entry point. Histochemical staining revealed that the spindle did not have a symmetrical structure along the equator, and this result does not agree with previous findings. Exploring the distribution and structural characteristics of muscle spindles in skeletal muscle can provide some anatomical basis for the study of muscle spindles at the molecular level and treatment of exercise-related diseases and provide a comprehensive understanding of muscle spindle morphology.

## Introduction

The muscle spindle, which is ubiquitous in skeletal muscle, is a proprioceptor that adjusts muscle tensile strength, velocity, and motive rate variation, and participates in the maintenance of muscle tension and the fine regulation of random movement across species (Banks, 2015). The morphological and electrophysiological variations of muscle spindles are essential for the assessment of motor function under pathophysiological conditions. However, the evaluation indicator was overlooked in many related studies for the scarce knowledge of muscle spindles in anatomy and morphology (Pang et al., 2014; Asano et al., 2019). Hence, related details in muscle spindles should be further explored. The number and distribution of muscle spindles differ between skeletal muscles with different functions, thus increasing the difficulty to understand the small organ (Osterlund et al., 2011). Studies using histochemical technology since 1960 by Barker, Lennartsson, Scott, and others revealed the muscle spindle distribution in different skeletal muscles both in mammals and birds (Lennartsson, 1980; Barker and Chin, 1987; Rowlerson et al., 1988). Given the distribution pattern of muscle spindles in the skeletal musculature across species, muscle spindle distribution is near nerve-entry points (NEPs) (Kokkorogiannis, 2004). Considering the variation in reporting and the lack of statistical analysis in many studies, the distribution specificity of muscle spindles in different skeletal muscles has not been explored.

The analysis of muscle spindles is time-consuming and prone to experimental failure caused by the improper handling of muscle tissue. Effective and convenient methods are still under development, and limited reports are available (Saverino et al., 2014). In 2017, the complete reconstruction of the special distribution of the soleus in C57BL/6 mice was obtained by synchrotron radiation−based computed tomography (SRCT). It presented the first 3D visualization and quantification of muscle spindles and their intrafusal fibers and the supplied neurovascular bundle in an intact muscle (Zeller-Plumhoff et al., 2017). However, the limited availability of SRCT equipment hindered the generalization of this research method (Fiorentino et al., 2020).

The same problem was encountered in the research of the single muscle spindle, which has been divided into three regions (i.e., regions A, B, and C) according to the intertwined nerve terminal ends and the connective tissue capsule. The structural integrity of the muscle spindle was ensured by checking whether all the cross-sections, where the muscle spindles were spotted, were not missing. A few studies have evaluated the morphology of the muscle spindle and performed related analysis of commonality. In 1984, Robert Bank, a British scientist, reconstructed the semi-schematic and axonometric architecture of a cat tenuissimus spindle from serial, semi-thin sections for defining motor innervation. However, the diagram can hardly be considered as the morphological index of one muscle spindle for the absence of the capsule and region C (Thornell et al., 2015). However, considering that the unprecedented, straightforward reconstruction was time-consuming, only three spindles were described, and relevant reports thereafter were limited. Although limited information is available, the muscle spindle can be considered to have an asymmetrical structure along the equator. In 2003, spindles in bovine calf extraocular muscle were 3D-reconstructed by an external marker-based automatic congruencing technique. However, considering the improper treatment of muscle tissues and the lack of nucleus disposing in the reconstructed description, the regions and equator of the spindle could not be distinguished, and the spindle’s symmetry could not be identified (Blumer et al., 2003). Considering that the asymmetrical structure of the muscle spindle has not been established, many researchers presumed a symmetrical anatomy along the equator (Sonkodi et al., 2020; Kroger and Watkins, 2021).

In the present study, we elaborate further on the distribution and morphological characteristics of muscles spindles in certain skeletal muscles. Improved ultra-low temperature frozen technology was utilized for skeletal muscle treatment, and continuous frozen cross-sections were obtained through a time-consuming process to analyze the muscle spindle location and the morphological characteristics in the tibialis anterior (TA), extensor digitorum longus (EDL), gastrocnemius (GA), and soleus muscle (SOL) in C57BL/6 mice. Our data showed the distribution heterogeneity of spindles across muscles, though the distribution generally corresponded to the pattern of near the NEPs. The asymmetry of the single muscle spindle was confirmed by statistical analysis.

## Materials and Methods

### Ethics Approval

All experimental protocols were approved by the Institutional Animal Care and Use Committees of Capital Medical University (ethical approval number AEEI 2018-153) and performed in accordance with the National Laboratory Animal guidelines (Ministry of Health, PR China, 1998). The authors understand the ethical principles under which the journal operates and this study complies with this animal ethics checklist.

### Animals

Five wide-type female C57BL/6 mice aged 4–5 months (weight 23–26 g) were purchased from Charles River (Beijing, China) and they were acclimatized to the Specific Pathogen Free mouse room for 1 week with a standard light/dark cycle. The temperature (23.5 ± 0.5°C) and humidity (40–50%) were well controlled. Food and water were always available in the cages.

### Organizational Preparation

All skeletal muscles were obtained from the lower extremities in mice. After administrating anesthesia by intraperitoneal injection of pentobarbital (1%, 50 mg/kg), normal saline was perfused into the heart to remove the blood and peel off skeletal muscles, and the mice were humanely euthanized. The muscles, including TA, EDL, GA, and SOL, were blotted to remove the surface moisture carefully, and embedded in OTC compound (Sakura, 4583). To fixed the isolated muscles at the physiological sarcomeric length, the rostral was fixed with a pin, while a slight stretch was held at the tendon in the caudal, and frozen immediately in melted 2-methylbutane (isopentane, Sigma, PHR1661) precooled in liquid nitrogen. To avoid cracks in skeletal muscle during freezing, we set the freezing time to less than 7 s. Then, samples were marked and stored at −80°C in a refrigerator for future use.

### Histochemical Staining

All skeletal muscles were taken out from −80°C and quickly transferred to the precooled constant cold box slicer (Leica, CM1900). The skeletal muscles were cryoprotected, and consecutive 10 μm-thick cross-sections were made. Importantly, during amelioration, the skeletal muscle tissue was kept in a frozen state, otherwise, it was extremely easy to form ice crystals. Continuous sections were stained with hematoxylin and eosin (H&E) to identify the general morphology and distribution of the muscle spindles. The following H&E routine steps were carried out: Frozen sections were recovered to room temperature and stained in hematoxylin for 3 min, color-separated with 1% hydrochloric acid and alcohol for 30 s, and treated with water for blue color return for 12 min. Then, the sample was stained in eosin for 1 min, dehydrated with gradient alcohol, and subjected to xylene clearance and subsequent neutral resin sealing. Finally, slides were digitally scanned using a Panoramic SCAN slide scanner (3D Histech, Hungary) with a 20 × or 40 × objective.

### Methods for the Analysis of Muscle Spindle Distribution and Dissymmetry

Considering that muscle spindles are related to NEPs, spindle distribution was assessed with NEPs as a reference. In the ventral-dorsal direction, the distance between NPE projection to muscle spindle (NM) or the most distal edge of muscles (NE) were measured in all muscles. The ratio of NM to NE, defined as the distance coefficient (DC), was used for the subsequent evaluation of spindle distribution. In the longitudinal pattern, each muscle was divided into three domains on average, and the NEP location was marked. To avoid double counting, from the appearance of the intrafusal fibers in certain cross-sections to the disappearance of intrafusal fibers in the continuous cross-sections, this was recorded as one muscle spindle. When calculating the distances between the spindles to NEPs, all the spindles’ equators were marked, and the spots were set as the position where the spindles were located. The distance between the equators and the NEP’s projection in the slide where the equator was located was then calculated. The density of muscle spindles was calculated according to the volume of the area and the spindle number (muscle spindle density = spindles number/the volume of the area). In this study, we set the NEPs at the place where the nerve enters skeletal muscle, regardless of the sub-branches after the nerve fiber entered muscles (i.e., in the case of the EDL, the deep peroneal nerve entered into the muscle at the cephalic end of its deep face, which was considered as the point of NEP, rather than the four sub-branches of the nerve fiber within the muscle). When analyzing if muscle spindles were symmetric along the equator, the spindle length was measured, and the absolute value of length differences in corresponding regions of two poles was calculated (0 was defined as symmetry) for subsequent analysis.

### Statistics

Statistical analysis was carried out using SPSS 20.0 software (IBM, San Diego, CA, United States). All data were tested for normality using the Kolmogorov–Smirnov test. The DCs of muscle spindles to NEPs in the dorsal-ventral distance and dissymmetrical analysis of spindles were performed using one-sample *t*-test with theoretical mean values of 0.5 and 0, respectively. The heterogeneity of muscle spindle distribution in the longitudinal perspective was determined by one-way analysis of variance with Bonferroni or Dunnett’s T3 multiple comparisons. Statistical significance was considered at *P* < 0.05. Graphs were generated using GraphPad Prism 8 software (GraphPad Software, San Diego, CA, United States).

## Results

### Muscle Spindle Distribution in Tibialis Anterior, Extensor Digitorum Longus, Gastrocnemius, and Soleus Muscle

To prove that the research on muscle spindles was accurate and reasonable, we first counted the muscle spindle number in each skeletal muscle. Spindle numbers in TA, EDL, GA, and SOL were counted by continuous frozen cross-sections. For the discrepant morphological characteristics of muscle spindles in different regions, the morphology of the surrounding extrafusal muscle fibers was used as a reference. The muscle spindle number and density in TA, EDL, GA, and SOL are shown in Supplementary Table 1. Similar results about TA, EDL, and SOL muscle spindle number have been observed in the literature. To our best knowledge, the spindle number of GA was directly counted for the first time in C57BL/6 mice.

**FIGURE 1:**
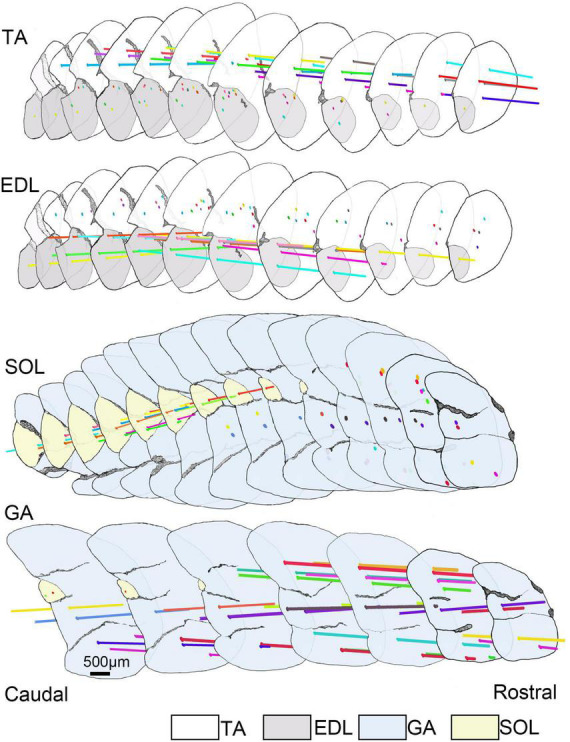
The 3D architecture of muscle spindles in TA, EDL, GA, and SOL. Selected sections (spaced by intervals of 600.00 μm) covering the central portion of the muscle length to reconstruct the 3D architecture of muscle spindles in TA, EDL, GA, and SOL. All selected sections (12 slides for TA/EDL, 14 slides for GA/SOL) were connected in series from caudal to rostral domains and the background was filtered. However, the outline of skeletal muscle and muscle spindle position was retained, and the anatomical information of muscle spindles was highlighted. The horizontal lines with different colors presented different muscle spindles, and the spots defined the location of muscle spindles in the cross-sections. TA, tibialis anterior; EDL, extensor digitorum longus; GA, gastrocnemius; SOL, soleus muscle.

To future explore the distribution pattern of muscle spindles in the skeletal musculature, we profiled the 3D architecture of muscle spindles in the four skeletal muscles combined with recorded parameters (Figure 1). The spindles were generally localized to focal regions within a transverse section. Muscle spindles were distributed in the whole muscle belly, and the abundance of spindles in the middle was higher compared with the two poles in TA, EDL, and SOL.

Further details about the muscle spindle distribution were obtained by analyzing the disposition of spindles in TA, EDL, GA, and SOL through plotting the relative distance between spindles and NEPs in dorsal-ventral and caudal-rostral directions, because NEPs are considered as parameters for evaluating the distribution trend in related research (Ovalle et al., 1999).

**FIGURE 2:**
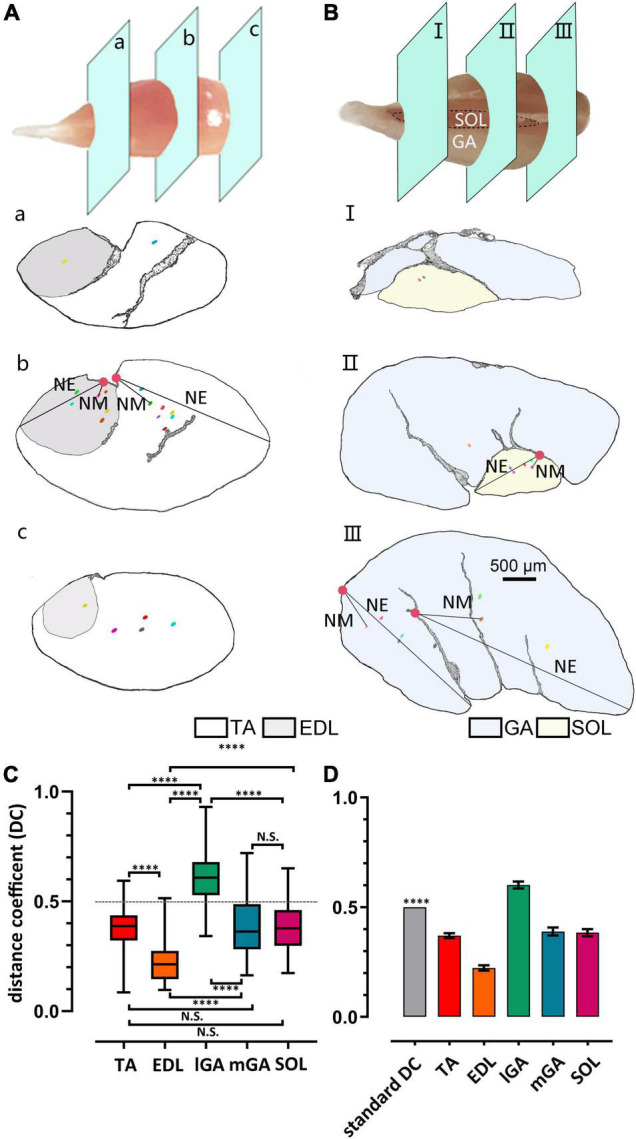
Heterogeneity of muscle spindle distribution in the ventral-dorsal direction across muscles. **(A,B)** Gross anatomy displayed the slides’ position displayed in caudal (a/I), middle (b/II), and rostral (c/III) domains in TA/EDL and GA/SOL, respectively. Muscle spindles were spotted by dots, NEPs were spotted by solid circles, and the representative distance between NPE to muscle spindle (NM) or the most distal edge of muscles (NE) was marked. **(C)** The difference in the DC index of different skeletal muscles. All muscle DC indexes showed significant difference, except when comparing TA and mSOL (*n* = 5). **(D)** DCs were significantly shorter than 0.5 in all muscles, except lGA, in which the spindles were not considered as close to the NEP. ^****^P < 0.0001. Error bars represent mean ± *SD*. TA, tibialis anterior; EDL, extensor digitorum longus; GA, gastrocnemius; lGA, lateral gastrocnemius; mGA, medial gastrocnemius; SOL, soleus muscle; NEP, nerve-entry point; DC, distance coefficient.

The frame diagram in Figure 2 characterizes the muscle spindles and NEPs (or their projected position) in caudal, middle, and rostral slides, in the dorsal-ventral direction. Considering the innervation differences between the medial gastrocnemius (mGA) and lateral gastrocnemius (lGA), the two parts were analyzed separately. Moreover, their corresponding H&E staining diagram and enlarged pictures of muscle spindles are exhibited in Supplementary Figures 1, 2.

Considering the inconsistently of the shape and size in skeletal muscles, a quantitative parameter is essential to assess the relationship between the muscle spindle and NEPs. In this study, a normalized index, the distance coefficient (DC), was established as follows:


$$\text{DC}=\frac{\mathrm{distance}\,\mathrm{of}\,\left|{\text{NEP}}_{\text{e}}-\mathrm{muscle}\,\mathrm{spindle}\,\mathrm{equator}\right|}{\mathrm{distance}\,\mathrm{of}\,\left|{\text{NEP}}_{\text{e}}-\mathrm{the}\,\mathrm{most}\,\mathrm{distal}\,\mathrm{edge}\,\mathrm{of}\,\mathrm{the}\,\mathrm{muscle}\right|}$$

NEP_*e*_ was set to the projection position of NEPs on certain slides in which the spindle equator was located. The distance between NEP_*e*_ and the spindle equator in all spindles was calculated.

According to the statistical results in Figure 2C and Supplementary Figure 3, muscle spindles showed larger DC (DC_*IGA*_ = 0.60 ± 0.12) in lGA than in other muscles (DC_*TA*_ = 0.37 ± 0.08, DC_*EDL*_ = 0.22 ± 0.09, DC_*mGA*_ = 0.39 ± 0.14 DC_*SOL*_ = 0.38 ± 0.12). However, no statistical difference was found between slow muscle SOL and TA and mGA (DC_*TA*_ vs. DC_*SOL*_, *P* = 0.51; DC_*mGA*_ vs. DC_*SOL*_, *P* = 0.80, respectively) on the observed parameter. Considering that DC > 0.5 (*P* < 0.0001, Figure 2D), the spindle distribution in IGA was not close to NEPs, at least in the dorsal-ventral direction. The distance between spindles and NEP in EDL was significantly closer than others although DC < 0.5 (*P* < 0.0001).

**FIGURE 3:**
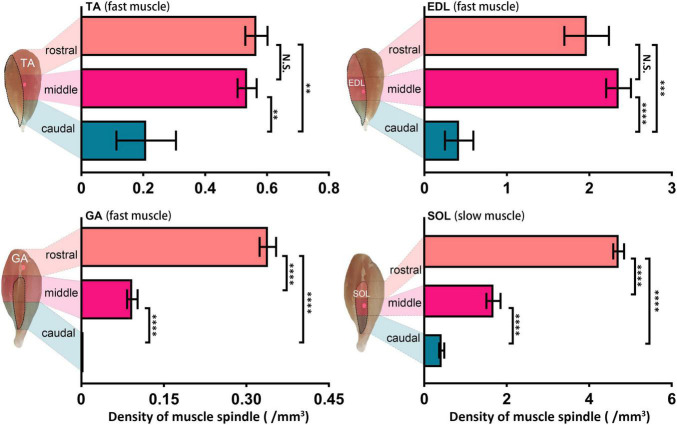
Muscle spindle distribution heterogeneity in the longitudinal pattern across muscles. Comparison of muscle spindle abundance in three caudal, middle, and rostral domains of TA, EDL, GA, and SOL (*n* = 5). The domain with the highest abundance of spindles was not consistent with the domain NEPs located in TA and SOL. ^**^P < 0.01, ^***^P < 0.001, ^****^P < 0.0001. Error bars represent mean ± *SD*. TA, tibialis anterior; EDL, extensor digitorum longus; GA, gastrocnemius; SOL, soleus muscle.

In a longitudinal perspective, the four muscles were divided into three domains, caudal, middle, and rostral, and the NEPs in their corresponding positions were spotted (Figure 3). Considering the close range and the same target in the same domain (caudal) of NEPs in lGA and mGA, the two NEPs were not marked in separate statistical analyses. The spindles in the three domains of the four muscles were numbered, respectively, and the spindle density in domains were calculated. Considerable differences were observed in the density of muscle spindles in diverse domains of one muscle (TAcau = 0.21 ± 0.01, TAmid = 0.54 ± 0.03, and TAros = 0.57 ± 0.04; EDLcau = 0.42 ± 0.17, EDLmid = 2.35 ± 0.15, and EDLros = 1.97 ± 0.27; GAcau = 0, GAmid = 0.09 ± 0.01, and GAros = 0.35 ± 0.03; SOLcau = 0.42 ± 0.06, SOLmid = 1.68 ± 0.17, and SOLros = 4.71 ± 0.13). Basically, the NEPs were located in domains with relatively higher spindle density than other domains. However, the domain with the highest spindle density was not consistent with the ones where an NEP was anchored in SOL. Moreover, the abundance of spindles in the caudal domain was significantly lower than the two other domains in all evaluated muscles (In TA, rostral vs. caudal, *P* = 0.002, middle vs. caudal *P* = 0.003; In EDL, rostral vs. caudal, *P* = 0.0002, middle vs. caudal *P* < 0.0001; In GA and SOL, all *P* < 0.0001), especially for GA, in which no spindles were observed.

### Muscle Spindle Dissymmetry

**FIGURE 4:**
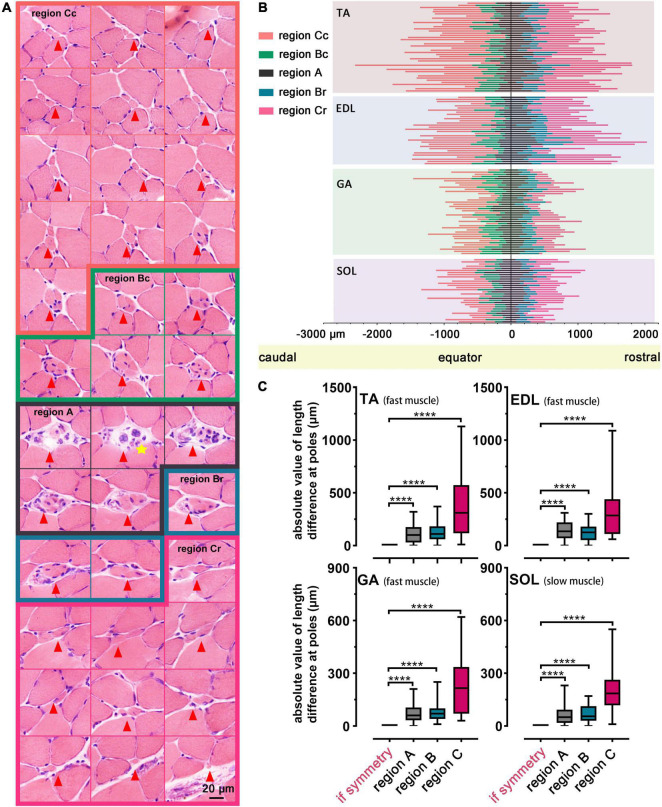
Dissymmetry of muscle spindles. **(A)** An overall view of a representative muscle spindle in TA was obtained by serial transverse sections (spaced by intervals of 100.00 μm). The regions of the muscle spindle, namely, region A (marked by black), region B (including region Bc and region Br, were marked by green and blue, respectively), and region C (including region Cc and region Cr, marked by orange and pink, respectively), in the longitudinal pattern were presented. A yellow star indicates the equator. Arrowheads mark the location of the muscle spindle in each slide. **(B)** Analysis of the dissymmetry along the equator, according to the length parameters of regions in 145 spindles of all muscles. Each muscle spindle was presented by a straight line, and the colors on each line were used to highlight different regions. Region Bc/Br (Cc/Cr) presented region B **(C)** in the caudal/rostral pole, and region A in poles was separated by a line marking the equator. **(C)** Statistical analysis revealed the dissymmetry of muscle spindles in TA, EDL, GA and SOL. ****P < 0.0001. Error bars represent mean ± SD. TA, tibialis anterior; EDL, extensor digitorum longus; GA, gastrocnemius; SOL, soleus muscle; region Bc/Br, region B in caudal/rostral domains; region Cc/Cr, region C in caudal/rostral domains.

Interestingly, the muscle spindle was not symmetric along the equator based on statistical analysis. An overall view of one muscle spindle was given by serial transverse sections, and every tenth slide (at 90 μm intervals) was subjected to exhibition, showing the dissymmetry of regions A, B, or C along the equator. In terms of the spindle shown in Figure 4A, within the slide marked with a yellow star, the nuclear bag fibers containing nuclei aggregated to clusters, representing the equator. The length of region C (naked without the capsule coating) was 1,500.00 ± 100.00 μm (marked with orange) in the caudal domain and 1,200.00 ± 100.00 μm (marked with pink) in the corresponding region in the rostral domain. Slides enclosed by green (500.00 ± 100.00 μm) and blue (300.00 ± 100.00 μm) portrayed region B (coated by capsule tightly) of caudal and rostral domains, respectively, while the remaining slides corresponded to region A, which was dissymmetric. And a schema diagram of a muscle spindle is displayed in Supplementary Figure 4.

To further explain the observed phenomenon, we listed 145 muscle spindles with intact dimensions to proceed to the demonstration in TA, EDL, GA, and SOL (Figure 4B). Different regions of spindles were represented with different colored lines, and the dissymmetry was further identified in corresponding regions on both sides along the equator (*P* < 0.0001, Figure 4C). No systematic bias in the asymmetry was identified by the *t*-test (Supplementary Figure 5).

The two parts at the poles of region C had a greater length difference than regions A or B, and this phenomenon might be related to the long dimension of region C. The difference between the corresponding regions at the poles of spindles did not depend on their location in the muscle. The muscle spindles in TA and EDL were slightly longer than those in GA and SOL (Figure 4B). The dimension of the spindle manifested the sensitivity of skeletal muscle to electrical signals moderately, and it could be regarded as a parameter to evaluate muscular function.

## Discussion

In the current study, a straightforward and time-consuming method was utilized, and the muscle spindle distribution in the lower extremity skeletal muscles of C57BL/6 mice were described through the reconstruction of their muscles and spindles in over 8,000 serial cross-sections. The findings provide insight into the spindle distribution characteristics through a systematic analysis of the relative distance between NEPs and spindles. Despite some heterogeneities in the spindle distribution of certain muscles in ventral-dorsal or caudal-rostral directions (Figures 2, 3), the results obtained support that the location of muscle spindles was close to NEPs based on comprehensive analysis. The results also indicate the specificity of spindle distribution across muscles, indicating the necessity to determine the anatomical characteristics when studying spindles. Some studies revealed that fast muscles possessed muscle spindles which were mainly concentrated around the NEPs, whereas in slow muscles they were evenly distributed throughout the muscle (Kokkorogiannis, 2004). Our results revealed that the muscle spindle distribution both in fast and slow muscles was associated with NEPs, which supported the view that “the distribution of muscle spindles is near to NEPs.”

Hitherto few studies reported that muscle spindle impairment was caused by hereditary factors in clinic. As the most important proprioceptor, however, many muscle and motor diseases, such as Parkinson’s disease (Conte et al., 2013), multiple sclerosis (Cameron et al., 2008), Huntington’s disease (Villalon et al., 2017), amyotrophic lateral sclerosis (Vaughan et al., 2015), and spinal muscular atrophy (Mentis et al., 2011) can cause the change of spindle morphologically and functionally. Recent studies on muscle spindles in the above diseases has advanced deeply to the molecular level. For example, in a study with amyotrophic lateral sclerosis, both sensory and motor neurons connected with intrafusal fibers accumulating misfolded SOD1 protein were reported to be responsible for the degeneration of annulospiral endings, loss of motor control, and ataxia (Vaughan et al., 2015).

With the development of biotechnology and the improved understanding in medical research, the muscle spindle has obtained considerable attention in multiple biological and medical fields because of its significant morphological changes and potential biological functions of such physiologic processes (Muller et al., 2008; Murgia et al., 2017). Anatomical observations indicated that rats with diabetes showed severe muscle spindle intrafusal fiber atrophy and gamma-motor nerve sparsity (Muramatsu et al., 2017). In adult rats, sciatic nerve axotomy leads to the atrophy of denervated muscles, including TA and EDL, with associated muscle spindle degeneration, and the spindle morphological and electrophysiological functions recovered after neural loop reconstruction (Elsohemy et al., 2009). Considering the limitations in anatomical research about spindles across skeletal muscles, existing research has overlooked this proprioceptor as an evaluation index.

Recent studies pointed out the positive effect of muscle spindles after incomplete spinal cord injury (SCI). Without muscle spindles (early growth response 3 knock out), the experimental mice were not able to promote incomplete SCI recovery (Macefield, 2013; Takeoka et al., 2014). The research pinpointed that such recovery relies on muscle spindle feedback that was necessary for neuronal circuit remodeling and motor control facilitation, but detailed knowledge is lacking to reveal the genetic regulation mechanism.

Considering that the remarkable characteristics of muscle spindles were uncovered, protecting the structural and functional integrity to the greatest extent possible is an effective treatment strategy for patients with long-term motor dysfunction (Tedeschi and Bradke, 2014). The level of neurotrophin-3 (NT-3) provided by afferent neurons may be a principal factor in determining the morphology and function of spindles in skeletal muscles of adults under normal physiological conditions (Oliveira Fernandes and Tourtellotte, 2015). Once the neuronal circuit was damaged, the deprivation of endogenous neurotrophic factors deteriorated muscle spindles, and the active exogenous injection of neurotrophic factors and electrical stimulation can compensate for them at least partially to resist spindle atrophy. Identifying the positional information of muscle spindles in skeletal muscles may accurately estimate the range for neurotrophic factor injection and electrical stimulation (Kim et al., 2009; Petruska et al., 2010; Gong et al., 2018). The characteristics of spindles located in mice skeletal muscles shown in this research were similar with those in cats previously reported (Matthews, 1972). Meanwhile, John et al. found that the spindles in the masseter were almost entirely concentrated in the anterior/deep part of the muscle in rodents, cats, and primates (Rowlerson et al., 1988; John, 2012). The distribution of muscle spindles might share similar characteristics across species, which indicates that our research might be used to explore the morphological basis and potential therapeutic methods to provide a reference for motor nerve loop injury repair and clinical treatment (Proske, 2008). In the present study, the statistical distribution of muscle spindles in the lower extremity skeletal muscle in C57BL/6 mice provided some anatomic parameters for estimating the lower extremity movement and related nerve injury and recovery.

However, the unclear distribution of muscle spindles hampered their filtration from extrafusal fibers under a light microscope, thus limiting the progress in understanding the genomics, transcriptomics, proteomics, and molecular biology of spindles. Information about the anatomical characteristics of muscle spindles helped in subsequent studies on development, function, and regeneration (Rigoni and Negro, 2020). In addition, the increasingly widespread use of transgenic mice provides new ideas and methods for the realm of basic scientific research. Muscle spindles or the connecting nerve fiber can be visualized by the expression of reporter proteins, such as green fluorescent protein in transgenic mice (Campsall et al., 2002). The advent of transgenic mice, together with advances in tissue clearing and high-resolution fluorescent imaging, will improve the understanding of the spatial distribution of muscle spindles. Once the new visualization methods were established, anatomical and morphological characteristics of muscle spindles could be clearly observed from the whole skeletal muscle, which made it possible to widely compare the consistency and heterogeneity of muscle spindles in different skeletal muscles. The parameters designed in this study may provide reference for the quantitative analysis of muscle spindle distribution in the future.

To explore some general characteristics of muscle spindles, we recorded some parameters for subsequent studies. Excluding incomplete muscle spindles, 145 muscle spindles with complete parameters were obtained in the follow-up analysis among the 310 muscle spindles that were counted. Another anatomic description of muscle spindles is that the dissymmetry along the equator emerged according to the intact dimension of 145 muscle spindles. Moreover, this asymmetry is consistent in both fast and slow muscles.

Considering that the symmetrical structure of the muscle spindle has not been established, many researchers have presumed a symmetrical anatomy along the equator. Two parts of the pole of regions were rarely equal in the majority of cases, indicating the absence of correlation with the location of the spindle in its muscle. Since the 1980s, the muscle spindle has been assumed to be asymmetrical, although after reconstructing the profile of the muscle spindle and based on morphological research on efferent fibers, the spindle has been accepted to be symmetrical (Jalaleddini et al., 2017). Considering the absence of related research, muscle spindle dissymmetry needs to be further explored.

The research aimed to determine the distribution pattern of muscle spindles in TA, EDL, GA, and SOL. Hence, the muscle spindle anatomical profile of other lower extremity muscles was beyond the scope of the current study. Generally, the distribution of muscle spindles is considered close to NEPs to interpret the spindles’ location, but other potential evaluation parameters can be used, such as Golgi tendon organ and myofiber type, which are beyond the scope of this study (Lund et al., 1978). These aspects should be further studied, and related experiments should be incorporated in future research.

## Conclusion

Our study involved the reconstruction of the 3D distribution of muscle spindles and uncovered the heterogeneity of the proprioceptor in TA, EDL, GA, and SOL. We considered muscle spindle distribution as an anatomic basis for the treatment of exercise-related diseases and the exploration of the physiological mechanism. The detailed dissymmetry and heterogeneity of spindles could help to understand the anatomical and morphological characteristics and evaluate the variation of spindles in pathophysiologic conditions in the future.

## Data Availability Statement

The original contributions presented in the study are included in the article/Supplementary Material, further inquiries can be directed to the corresponding author/s.

## Ethics Statement

The animal study was reviewed and approved by Institutional Animal Care and Use Committees of Capital Medical University.

## Author Contributions

XL and ZY: conception, design, and supervision of the study. WL: experimental operation and data analysis. WL, FH, and PH: data visualization and validation. YG and WZ: project administration. WL and J-SR: writing—original draft. J-SR and HD: writing—review. All authors revised the manuscript and approved its final version, and agreed to be accountable for all aspects of the work in ensuring that questions related to the accuracy or integrity of any part of the work are appropriately investigated and resolved, designated as authors qualify for authorship, and those who qualify for authorship are listed.

## Conflict of Interest

The authors declare that the research was conducted in the absence of any commercial or financial relationships that could be construed as a potential conflict of interest.

## Publisher’s Note

All claims expressed in this article are solely those of the authors and do not necessarily represent those of their affiliated organizations, or those of the publisher, the editors and the reviewers. Any product that may be evaluated in this article, or claim that may be made by its manufacturer, is not guaranteed or endorsed by the publisher.
