# Supplementary material for: Distribution Heterogeneity of Muscle Spindles Across Skeletal Muscles of Lower Extremities in C57BL/6 Mice
Source: Front Neuroanat. 2022 Mar 17;16:838951. doi: 10.3389/fnana.2022.838951 (PMC8968039; doi:10.3389/fnana.2022.838951)
Supplement: Supplementary file 1 [file Data_Sheet_1.pdf]

Table S1 muscle spindle density in mouse skeletal muscles

	skeletal muscle weight(mg)	muscle spindles Number	muscle spindles density[#]
TA	36.06±1.68	16.40±0.68	0.50±0.01
EDL	10.14±0.74	10.40±0.40	1.80±0.09
GA	143.1±1.04	23.40±0.81	0.15±0.01
SOL	8.82±0.33	11.00±0.84	1.53±0.09

muscle spindle density=spindle number/ the volume of muscle

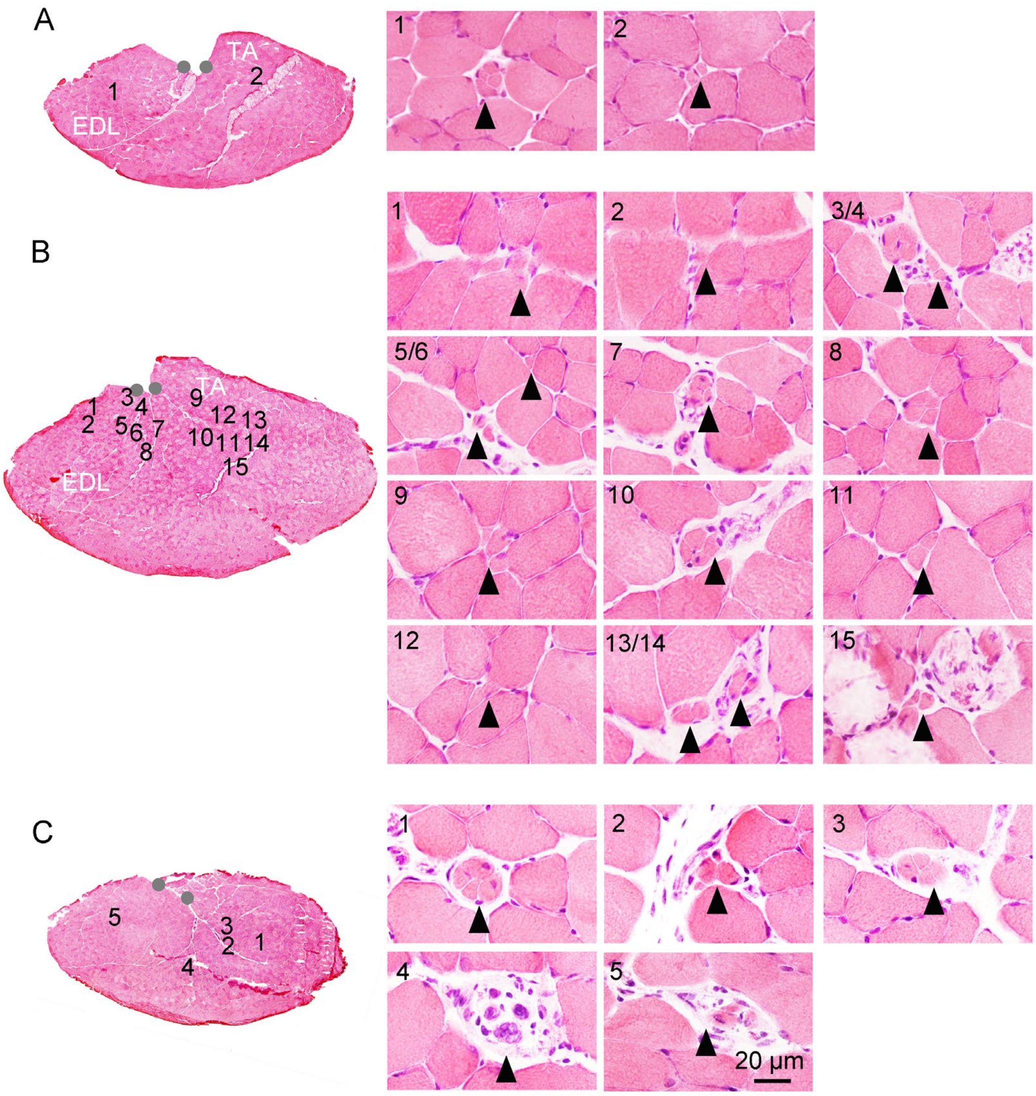

Figure S1 HE staining showed the distribution of muscle spindles in TA and EDL.

The muscle spindles distributions in caudal **(A)**, middle **(B)** and rostral **(C)**, corresponding to figure 2A, was described by HE staining. NEPs were spotted by solid circles. Arrowheads mark the location of the muscle spindle in slides. The enlarged pictures were corresponded to the numbered fields on the left. TA, tibialis anterior; EDL, extensor digitorum longus; NEP, nerve enter point.

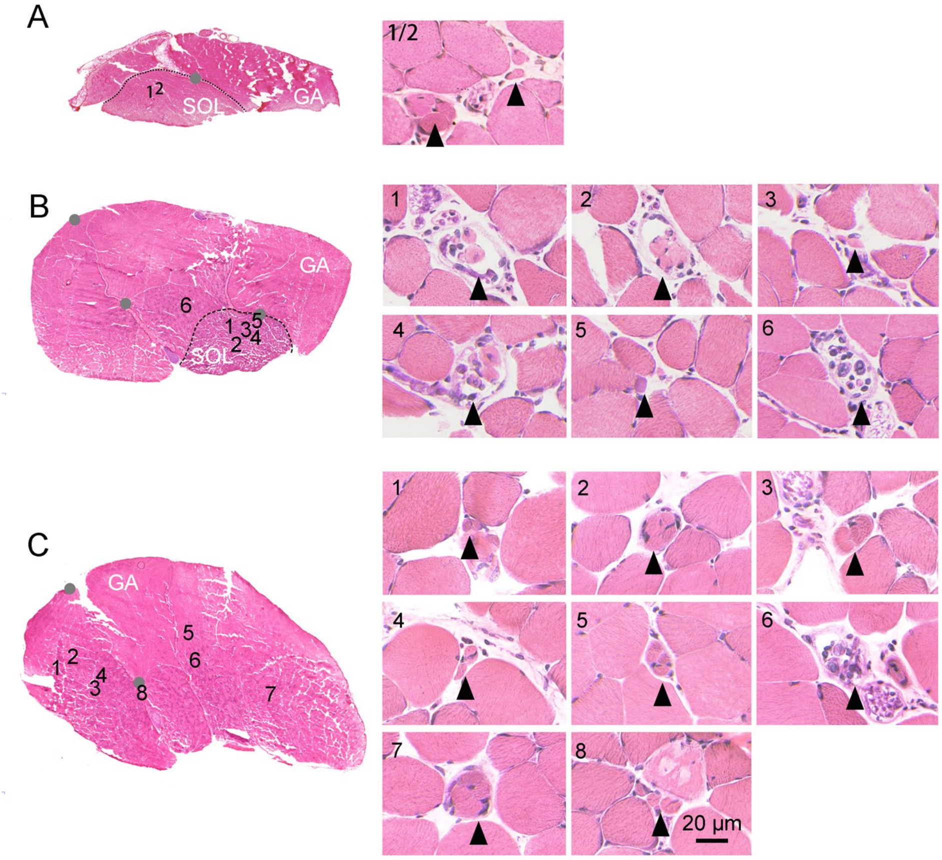

Figure S2 HE staining showed the distribution of muscle spindles in GA and SOL.

The muscle spindles distributions in caudal **(A)**, middle **(B)** and rostral **(C)**, corresponding to figure 2B, was described by HE staining. NEPs were spotted by solid circles. Arrowheads mark the location of the muscle spindle in slides. The enlarged pictures were corresponded to the numbered fields on the left. GA, gastrocnemius; SOL, soleus muscle; NEP, nerve enter point.

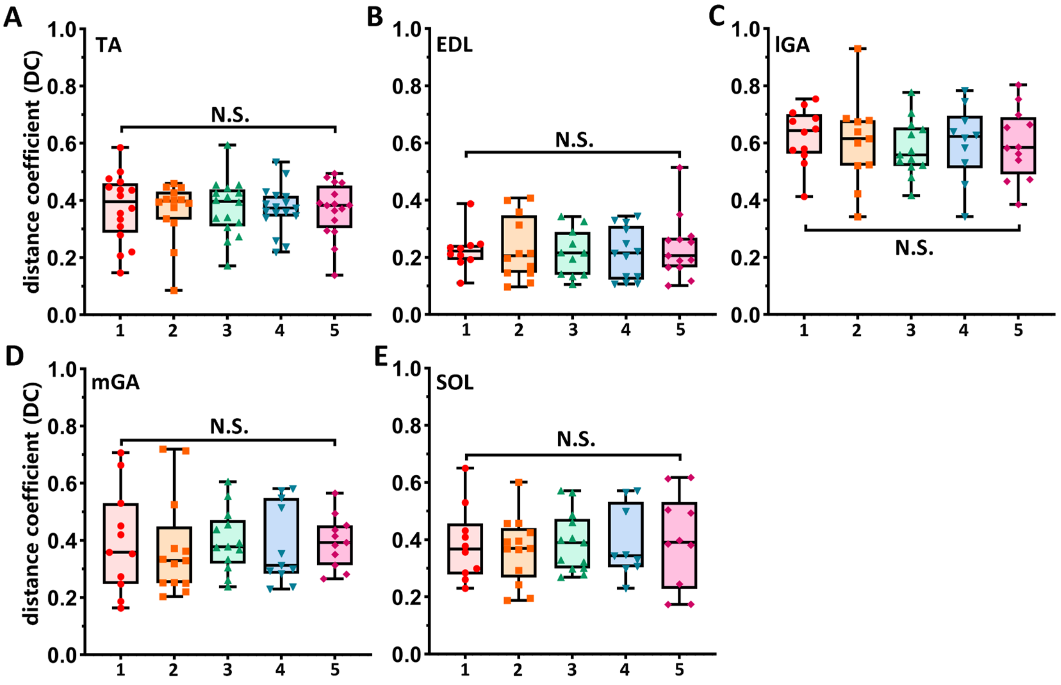

Figure S3 Individual differences in muscle spindle distribution in the ventral-dorsal direction.

The distance coefficient index in TA, EDL, lGA, mGA and SOL of five mice (#1-#5), respectively, and no significant difference was found among animals. Error bars represent mean ± SD. TA, tibialis anterior; EDL, extensor digitorum longus; GA, gastrocnemius; lGA, lateral gastrocnemius; mGA, medial gastrocnemius; SOL, soleus muscle.

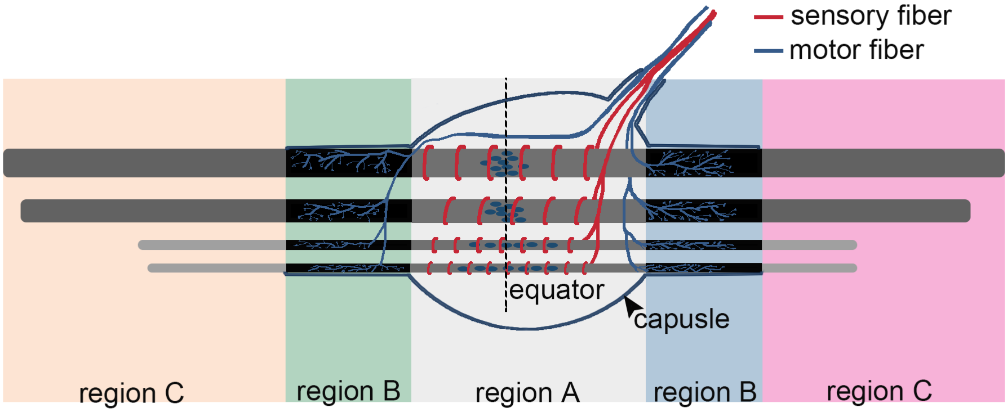

Figure S4 The schema diagram of a typical muscle spindle in mouse. The criteria of the subdivision of muscle spindles into A, B and C regions is as follows: the A and B regions are encapsulated, where the A region contains the equatorial region with a periaxial space, nuclear accumulation and the sensory innervation for all intrafusal fibers irrespective of type; the B region is the encapsulated polar zones with motor innervation; and the C region is the extracapsular portions of the spindle. (Banks and Barker, 2004; Thornell et al., 2015).

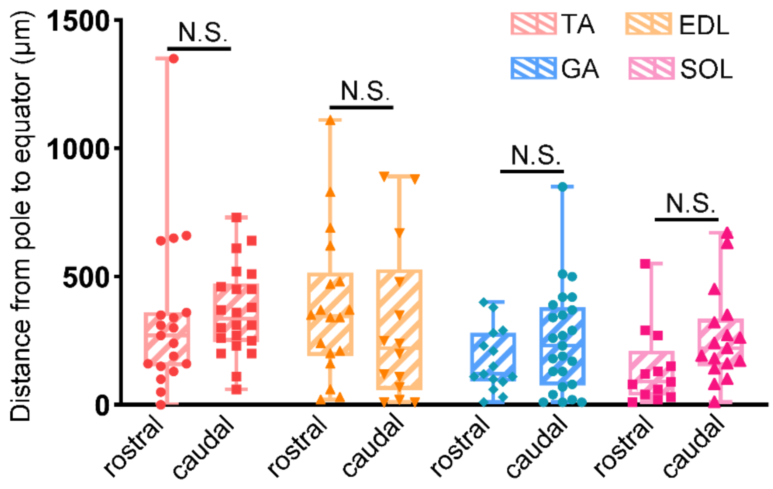

Figure S5 The muscle spindle dissymmetry showed no directional character.

The difference in length between the rostral and caudal sides of the spindles equator was calculated. No trend in muscle spindle asymmetry could be found.

References

Banks, R.W., and Barker, D. (2004) The muscle spindle. In: Myology. 3rd edn. New York: McGraw-Hill Press.

Thornell, L.E., Carlsson, L., Eriksson, P.O., Liu, J.X., Osterlund, C., Stal, P., and Pedrosa-Domellof, F. (2015). Fibre typing of intrafusal fibres. J Anat. 227, 136-156.
